# Lactic Acid Bacteria in Raw-Milk Cheeses: From Starter Cultures to Probiotic Functions

**DOI:** 10.3390/foods11152276

**Published:** 2022-07-29

**Authors:** Márcia C. Coelho, Francisco Xavier Malcata, Célia C. G. Silva

**Affiliations:** 1 Institute of Agricultural and Environmental Research and Technology (IITAA), University of the Azores, 9700-042 Angra do Heroismo, Portugal; marciacoelho8282@gmail.com; 2 FEUP—Department of Chemical Engineering, University of Porto, 4200-465 Oporto, Portugal; fmalcata@fe.up.pt; 3 LEPABE—Laboratory for Process Engineering, Environment, Biotechnology and Energy, Faculty of Engineering, University of Porto, 4200-465 Oporto, Portugal; 4 ALiCE—Associate Laboratory in Chemical Engineering, Faculty of Engineering, University of Porto, 4200-465 Oporto, Portugal

**Keywords:** cheese, LAB, bacteria, lactobacilli, bacteriocins, probiotics, health-promoting effects

## Abstract

Traditional cheeses produced from raw milk exhibit a complex microbiota, characterized by a sequence of different microorganisms from milk coagulation and throughout maturation. Lactic acid bacteria (LAB) play an essential role in traditional cheese making, either as starter cultures that cause the rapid acidification of milk or as secondary microbiota that play an important role during cheese ripening. The enzymes produced by such dynamic LAB communities in raw milk are crucial, since they support proteolysis and lipolysis as chief drivers of flavor and texture of cheese. Recently, several LAB species have been characterized and used as probiotics that successfully promote human health. This review highlights the latest trends encompassing LAB acting in traditional raw milk cheeses (from cow, sheep, and goat milk), and their potential as probiotics and producers of bioactive compounds with health-promoting effects.

## 1. Introduction

Lactic acid bacteria (LAB) are of great economic importance because they play an important role throughout the fermentation process of traditional cheeses when added accidentally or intentionally [1]. Their metabolic features not only contribute to the development of desirable sensory characteristics of food products but also allow the nutritional value of the raw material to be maintained or even enhanced [2].

The microbiota of raw milk cheeses are quite complex and include numerous strains of non-starter lactic acid bacteria (NSLAB), which are very important for cheese ripening and flavor development [3]. Since these cheeses have more intense and unique flavors compared to cheeses manufactured from pasteurized milk, there has been an increased interest in studying the functional and structural diversity of NSLAB. Several studies have attempted to comprehensively describe the microbiota in traditional cheeses at different stages of the ripening process [4,5,6,7,8,9,10,11]. Culture-dependent methods are most commonly used, but they are labor-intensive and inherently biased [12]. For this reason, the use of culture-independent techniques, as well as state-of-the-art sequencing techniques, have played a key role in the study of microbial populations in this type of cheese [5,13].

Such natural resources as traditional cheeses also represent some of the best sources of LAB strains useful to the food industry. Desirable properties of LAB for use as starter cultures and adjunct cultures in dairy products include good acidifying capacity, the ability to contribute to the desired flavor, and the possible production of exopolysaccharides (EPS) to improve texture [14]. LAB can also be used as protective cultures to control the number of contaminating or pathogenic microorganisms. Their probiotic potential is already considered a desirable feature of LAB. Hence, tolerance to gastrointestinal conditions, including resistance to gastric acidity, digestive enzymes, and bile salts, has been used as an indicator of the probiotic potential of LAB [15]. As recommended by FAO/WHO [16], in vitro tests to evaluate the probiotic potential of LAB also include adherence to mucus and/or human epithelial cells, antimicrobial activity against potential pathogens, and the ability to reduce adhesion of pathogens to surfaces.

Traditional cheeses are also useful in the isolation of LAB strains capable of producing bacteriocins, which reduce the risk of pathogen growth and survival [17]. The LAB in these cheeses may also have health-promoting potential, either by degrading nutrient-damaging compounds (e.g., biogenic amines and cholesterol) or by increasing the amount of beneficial compounds (e.g., antihypertensive peptides, γ-aminobutyric acid (GABA), short-chain fatty acids (SCFA), and conjugated linoleic acid (CLA)) [18,19]. For example, screening LAB for the ability to produce GABA has become relevant for use in fermented foods, as the inclusion of GABA as a food additive requires prior approval from the relevant authorities, and is not still allowed in some countries [19].

The aim of this review is to highlight the latest research on LAB—either deliberately added or naturally occurring in milk—their characterization, and their effects on the texture and flavor of raw milk cheeses. In addition, the health benefits of LAB as potential probiotics and producers of bioactive compounds are addressed.

## 2. Raw-Milk Cheeses

Cheese is found in almost all cultures, and is probably one of the oldest processed foods; some authors even suggest that it originated in Europe, ca. 7000 years ago [20].

**Figure 1:**
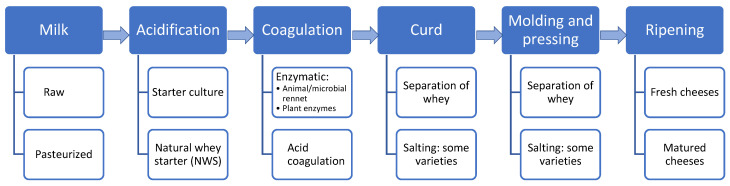
General protocol for cheesemaking process.

Cheese making is a complex process that involves the coagulation of milk, which can be enzymatic or acidic, thus resulting in a semi-solid curd composed mainly of casein and milk fat, followed by the syneresis and subsequent removal of the excess liquid (whey). The dehydration process, which concentrates milk fat and caseins, is controlled by a combination of techniques in addition to the biochemical composition of the milk. The moisture content, salt, pH, and microbiota of the cheese regulate and control the biochemical changes that occur during ripening and consequently determine the flavor, aroma, and texture of the final product [21]. Although the texture and quality of the finished cheese are strongly determined by the preceding processing steps, most characteristic aspects of texture and flavor actually develop during ripening; this explains the abundance of cheese varieties [22]. The production of cheeses generally follows a similar protocol (see Figure 1). However, several steps can be modified to obtain a product with the desired characteristics of each type/variety of cheese [23].

The indigenous microbiota of raw milk is usually quite diverse and heterogeneous and has a significant impact on the overall microbiota of cheese [24]. Some of these microorganisms, particularly LAB, may contribute to the acidification of milk during the initial steps of cheese making. In some artisanal cheeses, indigenous LAB from milk are used for the production of acid in the first steps of cheese making, without the need to add starter cultures [11]. To control the fermentation process, the technique of “back slopping” was developed, in which the whey of the fermented product is collected and stored to be used as inoculum for the next batch [23]. Variations of this method are still found in some traditional productions, where the whey from one day of cheese production is incubated and used as a starter culture on the next day [23]. Currently, most cheesemakers use a selected starter culture isolated from traditional cheese production [25].

The microbial sequence during cheese ripening is related to the ability of microbial populations to adapt to the specific environmental conditions that prevail in cheese [26]. The number of microorganisms present in ripened cheese depends on their ability to survive heat and acidity, to grow during ripening with energy sources other than carbohydrates present in milk, and to grow in and tolerate low water activity [27]. The quality of milk is also important for LAB growth, as toxic residues and contaminants affect its suitability for cheese making and the safety of the final product. The most common chemical residues found in milk are antibiotics administered to treat mastitis upstream, as they disturb starter cultures and NSLAB, and thus prevent milk acidification and normal cheese ripening [28]. Lytic bacteriophages are other spoilage agents that may be present in raw milk and affect cheese quality. Phages targeting key starter or adjunct cultures have been associated with changes in fermentation resulting in slow acidification and undesirable organoleptic characteristics of the cheese [24].

The sensory characteristics of cheese are also a consequence of the complex LAB community responsible for fermentation throughout the production process [29]. During cheese ripening, proteolysis is of great importance because it contributes to textural and sensory changes in the matrix of this product [30]. Such changes are the result of degradation products, such as peptides and even amino acids. The free amino acids resulting from proteolysis and the fatty acids released by lipolysis play an active role as substrates for a number of secondary reactions, which give rise to many important flavor compounds [31,32,33].

## 3. LAB Characterization

LAB have long been associated with food fermentation and preservation. Since they play multifunctional roles in numerous applications, they are considered the most important group of industrial microorganisms [34]. They comprise a heterogeneous group of genera that share many important physiological properties, such as the ability to ferment carbohydrates to lactic acid via homo- or heterofermentative metabolism [3].

LAB are characterized as Gram-positive, non-spore-forming, catalase-negative, cytochrome-deprived, and tolerant anaerobic bacteria [35,36,37]. They are fastidious, acid-tolerant, and have a strictly fermentative metabolism, with lactic acid being the major end product of sugar fermentation [35,38]. They have a low molar content of guanine + cytosine (G + C), are oxidase- and benzidine-negative, do not reduce nitrates to nitrites, are gelatinase-negative, cannot utilize lactate [39], and grow only in complex media [40]. The heterogeneity of this group is clearly expressed by their morphological characteristics; bacilli or cocci may also appear as single or grouped cells, namely tetrads and short or long chains [3].

The main characteristic of this group is its inability to synthesize porphyrin groups (e.g., heme); this explains the absence of cytochromes and “true” catalase in laboratory cultures. Under these conditions, considered normal for most studies of these bacteria, LAB lack the electron transport chain mechanism and rely on fermentation with substrate-level phosphorylation for energy production [41]. However, there are exceptions to this general rule, as some strains of LAB produce peroxidases or a “pseudocatalase”. In media containing hemoglobin or similar compounds, some strains can produce catalase or even cytochromes; in some cases, this results in respiration with a functional electron transport chain [35].

Due to their limited biosynthetic capabilities and high demand for carbon and nitrogen sources, the natural habitats of LAB are nutrient-rich environments. Therefore, LAB are usually associated with milk and its derivatives, meat and its derivatives, vegetables, beverages, soil, and sewage, and are also part of the respiratory, intestinal, and genital tract microbiota of humans and higher animals [35,40].

LAB cause the rapid acidification of fermented milk via the production of organic acids, mainly lactic acid [42,43]. During this fermentation, LAB inhibit the growth of most undesirable microorganisms by acidifying the environment; this is considered a fundamental characteristic of dairy products, especially in cheese production [3]. The metabolites produced during fermentation, with the exception of volatiles, remain in the food, and this helps inhibit the growth of undesirable bacteria. Properties favoring industrial application also include tolerance to various adverse environments, simple metabolism, and the ability to metabolize various carbon sources [34].

In addition to their capacity to produce lactic acid, LAB contribute to other product characteristics, such as flavor, texture, and nutritional value, as a consequence of their metabolic properties [3,42,43,44,45]. LAB metabolism leads to a variety of compounds, such as diacetyl, acetoin, and 2,3-butanediol from citrate utilization, as well as a wide range of volatile compounds and bioactive peptides from amino acid catabolism; they are known to affect the aroma and flavor of cheese [46].

Some LAB are also capable of producing metabolites with specific antagonistic and antibacterial activities, such as antifungal compounds and bacteriocins, which hold an enormous potential to inhibit various types of microorganisms [47]. The inhibitory properties of LAB depend on the species, pathogenic bacterium load, sanitary processes, and the amount of LAB in food [48]. Therefore, LAB can be used as a bioprotective culture to increase microbiological safety, extend shelf life, improve texture, and contribute to a pleasant sensory profile of the final product [42,43,47].

Due to their wide use in fermented products and a long history of safe human consumption, most LAB have Generally Regarded as Safe (GRAS) status, meaning that they are generally considered safe and have accordingly been approved by the U.S. Food and Drug Agency (FDA) [47,49,50,51]. In the European Community, qualified presumption of safety (QPS) status is granted by the European Food Safety Authority (EFSA).

LAB that are considered GRAS belong to the genera *Lactococcus*, *Oenococcus*, *Lactobacillus*, *Leuconostoc*, *Pedicoccus*, and some *Streptococcus* [51]. Species of the genus *Enterococcus* and some species of *Streptococcus* can be pathogenic and are therefore not eligible for GRAS status [49]. Due to safety concerns, no members of the genus *Enterococcus* are proposed for QPS status. The concerns associated with these bacteria arise from their virulence factors and resistance to a variety of antibiotics [52,53].

### LAB Taxonomy

The first classification of LAB was designed in 1919 by Orla-Jensen, who classified LAB into the genera *Betabacterium*, *Thermobacterium*, *Streptobacterium*, *Streptococcus*, *Betacoccus*, *Tetracoccus*, and *Microbacterium* [35]. The considerable changes that meanwhile took place in the taxonomy of LAB, involving the creation of new genera and species, and subsequent reclassification and restructuring thereof, have resulted in only the genus *Streptococcus* remaining from the original genera [35].

In current taxonomy, LAB belong to the phylum *Firmicutes*, the class *Bacillus*, the order *Lactobacillae*, and the families *Aerococcaceae*, *Carnobacteriaceae*, *Enterobacteriaceae*, *Lactobacillaceae*, *Leuconostocaceae*, and *Streptococcaceae* [54]. The family *Aerococcaceae* includes the genera *Aerococcus*, *Abiotrophia*, *Facklamia*, *Dolosicoccus*, *Eremococcus*, *Globicatella*, and Ignavigranum, and the family *Carnobacteriaceae* includes the genus *Carnobacterium* and other minor genera (*Alkalibacterium*, *Allofustis*, *Alloiococcus*, *Atopobacter*, *Atopococcus*, *Atopostipes*, *Desemzia*, *Dolosigranulum*, *Granulicatella*, *Isobaculum*, *Lacticigenium*, *Marinilactibacillus*, *Pisciglobus*, and *Trichococcus*) [55]. The family *Enterococcaceae* includes the genera *Enterococcus*, *Tetragenococcus*, and *Vagococcus*, and the smaller genera *Bavariicoccus*, *Catellicoccus*, *Melissococcus*, and *Pilibacter* [55]. The family *Lactobacillaceae* includes the genera *Lactobacillus* and *Pediococcus*, the family *Leuconostocaceae* includes the genera *Leuconostoc*, *Fructobacillus*, *Oenococcus*, and *Weissella*, and the family *Streptococcacea* includes the genera *Lactococcus*, *Lactovum*, and *Streptococcus* [35,54,55]. Recently, a merger of the families *Lactobacillaceae* and *Leuconostocaceae* has been proposed based on whole genome sequences and genome phylogeny [56].

LAB also include the only sporulated LAB, which belong to the genus *Sporolactobacillus* [57]. The genus *Bifidobacterium*, often considered in the same context as true LAB, shares some important typical features but is phylogenetically unrelated and belongs to the phylum *Actinobacteria* [34].

## 4. Main LAB Identified in Raw-Milk Artisanal Cheeses

Artisanal cheeses are often manufactured from raw milk in farms or small dairies, following specific protocols according to traditional heritage. They are distinguished by their flavor characteristics, and are generally associated to a particular region or country [29]. These cheeses have a complex microbiota characterized by the succession of different microorganisms throughout cheesemaking [58,59]. These microorganisms are an essential component of all ripened cheeses, and play an important role in cheese ripening by influencing the organoleptic and physicochemical characteristics of the final product [60].

Cheese produced from raw milk is often characterized by a richer and more distinctive flavor than its counterpart produced from pasteurized milk [61]. This difference results from the greater diversity of microorganisms in cheese produced from raw milk. The microbiota of raw milk includes *Lactococcus* spp., *Leuconostoc* spp., *Enterococcus* spp., *Streptococcus* spp., *Micrococcus* spp., *Staphylococcus* spp., *Arthrobacter* spp., *Corynebacterium* spp., *Brevibacterium* spp., *Enterobacter* spp., *Citrobacter* spp., and *Acinetobacter* spp.; they play a key role in the ripening and flavor development of cheese produced from raw milk [61]. Microbial succession during cheese ripening relates to the ability of microbial populations to adapt to specific environmental conditions that affect cheese characteristics [26].

The recent application of high-throughput DNA sequencing (HTS) supports a detailed analysis of the composition and functional potential of the microbiota of traditional raw milk cheeses. Bacterial communities differ among raw milk cheeses depending on the manufacturing process, but the bacteria in the cheese core are dominated by LAB belonging to the genera *Lactococcus*, *Lactobacillus*, *Enterococcus*, *Streptococcus*, and *Leuconostoc* [4,7,8,60,62,63,64].

### 4.1. Lactococcus

Bacteria of the genus *Lactococcus* are characterized by being Gram-positive cocci that occur singly, in pairs, or in a chain, are non-spore-forming, non-motile, facultatively anaerobic, non-β-hemolytic, and catalase-negative, and grow at 10 °C and at 40 °C, but not at 45 °C. They generally grow at 4% (*w*/*v*) NaCl, except *Lc. lactis* subsp. *cremoris*, which tolerates only 2% salt (*w*/*v*) NaCl [39,65]. They have a fermentative metabolism, with L-lactic acid being the predominant end-product of glucose fermentation via the glycolytic pathway [39,65,66].

This genus consists of 17 species, including *Lc. lactis* (subspecies cremoris, lactis, hordniae, and tructae), *Lc. garvieae*, *Lc. plantarum*, *Lc. raffinolactis*, *Lc. piscium*, *Lc. chungangensis*, *Lc. fujiensis*, *Lc. taiwanensis*, *Lc. hircilactis*, *Lc. nasutitermitis*, *Lc. petauri*, *Lc. formosensis*, *Lc. reticulitismitis*, *Lc. laudensis*, *Lc. termiticola*, and *Lc. allomyrinae* [67,68,69]. *Lc. lactis* is the species most commonly found in raw milk and dairy products [69,70].

Lactococci are mainly used as starter cultures for dairy products [39]. For example, *Lc. lactis* subsp. *lactis* and *Lc. lactis* subsp. *cremoris* are the main lactococci used as starter cultures for various cheeses [71]. These lactococci were selected for their metabolic stability, resistance to bacteriophages, and ability to produce unique compounds, many of which are derived from amino acid degradation [43].

### 4.2. Lactobacillus

The genus *Lactobacillus* comprises a large number of different species (261 species in March 2020) [56], with a relatively high degree of diversity [43,72]. The technologically and commercially most important species include *L. acidophilus, L. casei, L. delbrueckii, L. plantarum, L. rhamnosus,* and *L. salivarius* [71]. Recently, a reclassification of the genus *Lactobacillus* into 25 genera was proposed based on genome phylogeny and ecological and metabolic properties [56]. Zeng et al. [56] actually proposed 23 new genera, named *Holzapfelia, Amylolactobacillus, Bombilactobacillus, Companilactobacillus, Lapidilactobacillus, Agrilactobacillus, Schleiferilactobacillus, Loigolactobacilus, Lacticaseibacillus, Latilactobacillus, Dellaglioa, Liquorilactobacillus, Ligilactobacillus, Lactiplantibacillus, Furfurilactobacil-lus, Paucilactobacillus, Limosilactobacillus, Fructilactobacillus, Acetilactobacillus, Apilactobacillus, Levilactobacillus, Secundilactobacillus*, and *Lentilactobacillus.* The generic term ‘lactobacilli’ has been proposed to refer to all organisms classified as *Lactobacillaceae*.

Lactobacilli are the dominant bacteria in many fermented foods such as meat and dairy products and interact with the microbiota of the gastrointestinal tract when ingested [50]. Studies conducted on traditional raw milk cheeses describe the dominance of lactobacilli throughout food ripening [62].

Lactobacilli are characterized by being Gram-positive, non-spore-forming, catatase-negative bacilli or coccobacilli [73,74]. They are strictly fermentative, aerotolerant or anaerobic, and have complex nutrient requirements (e.g., for carbohydrates, amino acids, peptides, fatty acid esters, salts, nucleic acid derivatives, and vitamins) [75]. With glucose as a carbon source, lactobacilli can be homofermentative and produce more than 85% lactic acid, or heterofermentative and produce lactic acid, CO_2_, ethanol, and/or acetic acid in equimolar amounts [75]. Lactobacilli are therefore classified into three groups based on their fermentation characteristics [66,75,76,77]:

Group I: obligate homofermentative lactobacilli. Hexoses are almost exclusively (>85%) fermented to lactic acid via the Embden–Meyerhof–Parnas pathway (EMP). The organisms have fructose-1,6-bisphosphate aldolase, but no phosphoketolase, and thus pentoses and gluconate are not fermented. This group includes the species *L. acidophilus, L. delbrueckii*, and *L. salivarius*;

Group II: facultative heterofermentative lactobacilli. Hexoses are fermented to lactic acid almost exclusively via the Embden–Meyerhof–Parnas pathway (EMP). The organisms possess both aldolase and phosphoketolase, and therefore ferment not only hexoses but also pentoses. In the presence of glucose, the enzymes of the phosphogluconate pathway are inhibited. This group includes many of the lactobacilli found in ripened cheese (e.g., *L. casei, L. paracasei, L. plantarum*, and *L. curvatus*);

Group III: obligate heterofermentative lactobacilli. They have phosphoketolase but not aldolase and therefore ferment sugars in a heterofermentative manner. Hexoses are fermented via the phosphogluconate pathway, producing lactate, ethanol (acetic acid), and CO_2_ in equimolar amounts. Pentoses enter this pathway and can also be fermented.

Many lactobacilli are used in food production and preservation because they can acidify and/or improve the taste, texture, and nutritional value of foods [78]. Their natural habitat is very diverse, as these bacteria are found in virtually all environments where carbohydrates are available, from food, plants, and wastewater, to the oral, genital, and gastrointestinal tracts of humans and animals [74,78]. Most species are also part of the commensal gut microbiota of humans and animals [79]. Some lactobacilli are considered probiotics due to their beneficial effects on host health [72,78].

### 4.3. Enterococcus

Enterococci are Gram-positive cocci that occur singly, in pairs, or in short chains, and are facultative anaerobes [39,74,80]. They do not form spores, are mobile, and have a homofermentative metabolism, in which the end product of glucose fermentation via the glycolytic pathway is L-lactic acid. They are catalase-negative, but some strains produce a pseudocatalase and have high nutrient requirements [80]. Enterococci are also salt- and heat-tolerant and can generally grow in the presence of 6.5% NaCl at temperatures between 10 °C and 45 °C [39,66]. They can also grow at a pH of 9.6 and in the presence of 40% bile [81].

Enterococci are found in a variety of environments, including soils, surface water, sewage, plants, and the gastrointestinal tract of humans and animals [74,81]. Enterococci are also commonly found in large numbers in dairy products, especially cheeses produced from raw milk, with *E. faecalis* and *E. faecium* being the predominant species [70,81,82].

Unlike lactococci, enterococci are not completely eliminated by pasteurization and may therefore be present in large amounts in many cheeses [66]. It is generally believed that the presence of enterococci is due to poor sanitary conditions during processing. Although several strains have biochemical properties that mean that they are useful for technological applications, their utilization has been questioned because they are also used as indicators of fecal contamination of foods. Some species, such as *E. faecalis*, are promiscuous and can easily acquire antibiotic resistance genes, such as vancomycin, from plasmids or transposons [66]. Some strains of enterococci have been identified as potential pathogens, so the presence of virulence factors and resistance to certain antibiotics should be carefully evaluated before using them [82,83,84,85].

### 4.4. Streptococcus

All species of the genus *Streptococcus* are Gram-positive cocci that may be spherical or oval and are typically arranged in chains or pairs. They are also immobile, and do not form spores. Most streptococci are facultative anaerobes, but some strains require CO_2_ for growth. They are chemoorganotrophic, ferment carbohydrates to produce lactic and other acids, have complex nutrient requirements, and are catalase-negative [41,86,87]. They are moderately thermophilic [43], and tolerate less than 2% NaCl [39]. *S. salivarius* subsp. *thermophilus* grows at 45 °C but not at 10 °C, and grows in the presence of 2.5% NaCl but not at 4% [66].

The genome of *S. salivarius* subsp. *thermophilus* is 1.8 Mb in size, making it one of the smallest genomes of all LAB [43]. In addition, plasmids play a relatively insignificant role in this species [43].

Streptococci are an important component of the commensal microbiota of humans and animals and colonize the mucous membranes of the mouth, respiratory tract, gastrointestinal tract, and genitourinary tract. Some species are also found on the skin, and others can be isolated from foods such as milk and dairy products [86].

One of the main characteristics of streptococci is their ability to produce various types of hemolysis in media containing blood. The production of complete hemolysis zones (β-hemolysis) by some streptococci is an indicator of the presence of potentially pathogenic streptococci [87]. In some species, the appearance of α-hemolytic zones (partial hemolysis) around aerobically grown colonies may be due to the production of hydrogen peroxide [41,86].

Some strains of streptococci are pathogenic to humans, such as *S. pneumoniae, S. pyogenes*, and *S. agalactiae. S. salivarius* subsp. *thermophilus* has differentiated itself from other streptococci, and occupies a well-defined place in the ecological niche of milk [43]. This species is therefore widely used as a starter culture for the production of fermented foods due to several biochemical properties that include sugar and protein metabolism, exopolysaccharide synthesis, and flavor formation [43].

### 4.5. Leuconostoc

*Leuconostoc* are characterized as Gram-positive cocci, with irregular morphology that may be elongate or elliptical. Most strains appear in the liquid medium as single cocci, in pairs or in short chains [43]. However, cell morphology can vary depending on growth conditions: When bacteria grow in a glucose medium or in a solid medium, they are elongated, whereas most strains form ovoid cells when they grow in milk [43,88,89].

*Leuconostoc* species are facultative anaerobes, intrinsically resistant to vancomycin and do not hydrolyze arginine [39,90]. All species require a medium rich in complex growth factors and amino acids and exhibit slow growth and low acidification capacity [43,88,89,91]. Leuconostocci are immobile, do not form spores, and lack catalase and cytochromes [41,43,90,92]. They are heterofermentative and produce D-lactate, ethanol, CO_2_, and small amounts of acetate from glucose metabolism via the phosphoketolase pathway [41,43,66,90,93]. Other metabolic pathways include the conversion of citrate to diacetyl and acetoin, and the production of dextrans from sucrose [43,94].

Leuconostoc have complex nutritional requirements and are found in plants, dairy products, meat, and various fermented foods [88]. *Ln. mesenteroides* subsp. *mesenteroides* and *Ln. lactis* are the dominant *Leuconostoc* in milk and fermented dairy products. *Ln. mesenteroides* subsp. *cremoris* and *Ln. paramesenteroides* are less frequently detected in milk, probably due to their slow growth under psychrotrophic conditions [88].

*Leuconostoc* spp. play an important role in altering the organoleptic quality and texture of food products, such as milk, butter, cheese, and meat [88,90]. Because they are obligate heterofermenters, the production of CO_2_ may alter texture and cause late blowing in certain cheeses, although this often leads to moderate “eye” formation in cheese [88,95]. In addition, *Leuconostoc* are used as flavor formers in mixed starter cultures, such as *Ln. mesenteroides* subsp. *dextranicum*, and *Ln. mesenteroides* subsp. *cremoris* [39]. Certain strains also produce diacetyl and acetoin from citrate, contributing to the typical aroma and flavor of dairy products [88,91,93].

## 5. LAB as Starter Cultures

The bacteria most commonly used as starter cultures in cheeses are LAB [96]. The chief role of these cultures is to acidify the milk, and thereby inhibit the growth of other (undesired) bacteria [84,97,98,99]. The starter bacteria must produce enough acid to lower the pH of the milk to below 5.3 within 6 h at 30–37 °C, depending on the type of cheese [97,100]. The production of acid in the right amount and at the right time is a crucial factor to obtain high-quality cheeses [27,66,101]. Therefore, the ability of LAB to produce acid rapidly is one of their most important technological features [26]. The temperature during production, salt levels, and humidity should be controlled to ensure that the activity of starter cultures is sufficient to rapidly reach the targeted pH [97]. Starter cultures should also promote a sustainable environment in the cheese in terms of redox potential, salinity, and moisture that allows suitable rennet enzyme activity and the growth of the secondary microbiota [23,97,102]. Starter bacteria are undoubtedly the main players in the first hours of cheese production. However, from the 18th day to the 25th day of ripening, the number of these bacteria decreases drastically as a consequence of the decrease of lactose as a nutrient and their own autolytic behavior [27].

In addition to acid production during the fermentation process, starter cultures also contribute to cheese ripening since their enzymes are involved in the proteolysis, lipolysis, and conversion of amino acids into compounds that directly contribute to the flavor of the final product [97,100,101,103]. In addition, the use of starter cultures ensures microbiologically safe products, because these cultures inhibit the development of undesirable microorganisms by producing compounds that prevent their growth, such as organic acids, bacteriocins, and hydrogen peroxide [104,105,106].

The most commonly used starter cultures are members of the genera *Lactococcus, Lactobacillus, Streptococcus, Leuconostoc*, and *Enterococcus* [97]. Currently, *Enterococcus* is not granted this qualification due to regulations related to the qualified presumption of safety (QPS). However, some well-characterized strains continue to be used as starter cultures, co-cultures, or protective cultures in the food industry owing to their beneficial properties [107]. The most commonly used species in cheese production are *Lc. lactis, S. salivarius* subsp. *thermophilus*, *L. helveticus*, and *L. delbrueckii* [102].

At the beginning of production, LAB may be present as a native component of the milk, as happens with many artisanal raw milk cheeses [97]. In these cheeses, the spontaneous fermentation of the milk is driven by the development of the aforementioned microbiota. However, the outcome of such processes is unpredictable, as the physiological stage and extent of inoculum are beyond operator’s control [95].

Conversely, starter cultures are intentionally added and previously selected based on their effect upon fermentation and the desired properties of the product. The selection criteria vary, but the dominant criterion is usually the acidification rate at a given temperature, and the insensitivity to phages [23]. Handling characteristics and stability during production are also criteria for starter culture selection [108].

The proper selection of starter cultures and the characterization of each strain is very important to obtain products with reproducible organoleptic and structural properties by the end of cheese production [23,84,98]. By controlling the fermentation process, the said cultures reduce the variations in organoleptic quality and microbiological stability observed in cheeses without them.

### 5.1. Type of Starter Cultures

Starter cultures can be categorized as mesophilic or thermophilic, depending on the incubation and manufacturing temperatures at which they are used [98]. Mesophilic starter cultures have an optimal growth temperature of ca. 30 °C, while thermophilic starter cultures grow best between 40 and 45 °C [104]. Mesophilic and thermophilic cultures can be divided into defined and undefined cultures [97].

#### 5.1.1. Mesophilic and Thermophilic Starter Cultures

The starter cultures most commonly used in the production of fermented dairy products belong to the genera *Lactobacillus* and *Streptococcus*, namely the species *S. salivarius* subsp. *thermophilus, Lb. helveticus, Lb. delbrueckii* subsp. *Lactis*, and *L. delbrueckii* subsp. *bulgaricus* [104,105,109,110].

Mesophilic starter cultures include mainly the genera *Lactococcus* and *Leuconostoc* [105,110]. The LAB most commonly used as mesophilic starter cultures are *Lc. lactis*, including subspecies *lactis* and *cremoris* for being good acid producers [109,110]. Other mesophilic starter cultures include the species *Ln. lactis* and *Ln. cremoris* [109]. Mixed mesophilic cultures are usually 90% acid producers and 10% aroma producers [110].

In the production of hard cheeses, mesophilic starter cultures are predominantly used (e.g., *Lactococcus* spp.), although thermophilic cultures may also be used (e.g., *S. salivarius* subsp. *thermophilus*) [102].

#### 5.1.2. Defined and Undefined Starter Cultures

Starter cultures are usually composed of different species, or multiple strains of one species. Starter cultures can be divided into defined and undefined cultures [23,105,109]. The former usually consist of one or more strains with known characteristics [95]. They have usually been isolated from mixed cultures and selected based on important characteristics such as phage resistance, acid production, citrate utilization, and aroma and flavor formation [26,111]. Undefined starter cultures have partially known or all unknown species and strains in their composition [110].

Starter cultures used in the production of cheese can be divided into: (1) defined cultures with multiple strains (e.g., *Lc. lactis* subsp. *lactis* and *Lc. lactis* subsp. *cremoris* in Camembert and Brie cheeses); (2) defined cultures with a single strain (e.g., *S. thermophilus* in Mozzarella cheese); (3) defined mixed cultures (e.g., *S. thermophilus*, *Lb. helveticus*, *Lb. delbrueckii* subsp. *lactis*, *Lb. delbrueckii* subsp. *bulgaricus* and *Propionibacterium shermanii* in Emmental and Gruyere cheeses); and (4) undefined mixed cultures (e.g., whey starter in Italian cheeses such as Parmigiano Reggiano) [23,27,110,112].

For instance, the cultures used for the production of Gouda cheese were isolated from an undefined starter culture traditionally used for the production of this cheese, consisting of *L. lactis* subsp. *cremoris*, *L. lactis* subsp. *lactis* biovar diacetylactis, and *Ln. mesenteroides* [95,112].

#### 5.1.3. Natural Whey Starter (NWS)

Natural whey starter cultures (NWS) consist of an undefined culture of LAB and are mostly acid producers [113]. This type of starter is commonly used in the production of traditional artisanal cheeses using the back-slopping technique, which requires the inoculation of milk with whey or fermented milk from the previous day [26].

## 6. LAB as Adjunct Cultures

### 6.1. Selected Adjunct Cultures

Adjunct cultures can be defined as those added to cheese for purposes other than acid production, even though they often consist of microorganisms derived from ingredients (raw milk) or the cheese-making environment [97]. Adjunct cultures, selected from adventitious LAB, also called non-starter LAB (NSLAB), can therefore be added with starter to accelerate the ripening process and produce the desired flavor [110]. These cultures are selected to survive cheese curd cooking temperatures and participate in flavor development at a later stage of cheese ripening. Mesophilic cultures such as *L. casei* and *L. paracasei* are traditionally added with the starter to improve the flavor of dairy products [110]. They can also mitigate defects caused by contaminating adventitious LAB by inhibiting their development [84].

### 6.2. Natural Adjunct Cultures

Natural adjunct cultures are often adventitious cultures LAB, which are not part of the added starter culture [114,115]. Such adventitious LAB are usually difficult to grow in milk and do not contribute to acid production [97], but are critical for the final flavor and texture of the cheese [102,105]. These bacteria can grow with energy sources other than lactose, and are more resistant to environmental stress [27,116]. Adventitious LAB are present at very low concentrations in the curd but their populations start to increase during the first months of ripening and eventually become the dominant microbiota of longer ripened cheeses [3,26,27,116].

The composition of adventitious LAB varies depending on cheese type, the mode of processing, and ripening time [84,114,117,118]. The development of adventitious LAB during cheese ripening can be attributed in part to their ability to utilize available nutrient sources [26]. As lactose is metabolized during the first weeks of ripening, adventitious LAB can obtain energy from compounds such as lactic acid, citric acid, ribose, fatty acids, glycerol, and amino acids [26,119]. Because LAB possess a variety of hydrolytic enzymes convenient for cheese proteolysis and lipolysis, they are able to grow and act during cheese ripening [84,116,117,120].

### 6.3. Charaterization of Adventitious NSLAB

The adventitious NSLAB are a particularly heterogeneous group, and include mesophilic lactobacilli, enterococci, pediococci, and *Leuconostoc* [100,114,115]. Mesophilic lactobacilli are the predominant and most important group in the microbiota of NSLAB [121]. Among the mesophilic lactobacilli, facultative heterofermenters are the most abundant in NSLAB [114], mainly *L. casei* subsp. *casei*, *L. casei* subsp. *pseudoplantarum*, *L. paracasei* subsp. *paracasei*, *L. plantarum*, *L. rhamnosus, L. curvatus* [97,116], and *L. pentosus* [102]. The obligate heterofermentative species commonly found in cheese are: *L. fermentum, L. buchneri, L. parabuchneri*, and *L. brevis* [102], although other species of facultative or obligate heterofermentative lactobacilli also occur [116].

The most common pediococci found in cheese are *Pediococcus acidilactici* and *P. pentosaceus* [97]. Among enterococci, *Enterococcus durans*, *E. faecalis*, and *E. faecium* are most abundant in cheese [102,122]. Within the genus *Leuconostoc*, the species *Ln. mesenteroides, Ln. peseudomesenteroides*, and *Ln. citreum* have been detected in artisanal cheeses produced from raw milk [122,123,124,125].

The origin of NSBAL can vary, but the main source is raw milk [114,115,121] and, to a lesser extent, whey used as starter—NWS [121]. The microbial diversity of raw milk cheeses depends on the microbiota of the milk, the ingredients utilized, and the processes used in cheese production [114,115,116,121]. The cheese processing environment can also be a potential source of NSBAL, especially in the case of mesophilic bacteria, which can survive the processing environment and on the equipment itself, even after cleaning and disinfection, due to their ability to form biofilms [26].

NSBALs are generally associated with raw milk but are also present in cheese produced from pasteurized milk. The presence of NSBAL in cheese produced from pasteurized milk is due to airborne contamination, contact with equipment and/or ingredients used in cheese making, or thermoduric strains that survive pasteurization [120].

When artisanal cheese is produced without direct inoculation with starter cultures, the microorganisms involved in fermentation are derived from starting material and environmental sources [126]. Therefore, the inherent and unique flavors known in cheeses produced from raw milk are the result of a diverse indigenous microbiota [26]. These NSBALs dominate the microbiota of many aged cheeses and play a key role in the development of flavor and aroma throughout ripening. For instance, they contribute to the release of small peptides and amino acids, which in turn can be converted into alcohols, aldehydes, esters, and sulfur compounds that are associated with specific flavors and aromas of the ripened cheese [84].

## 7. Antimicrobial Activity of LAB

LAB can be used to inhibit or destroy undesirable microorganisms in foods, increase their safety, and extend their shelf life [127]. The use of LAB as bioprotective agents also ensures food quality and safety without the need to resort to chemical preservatives [128].

### 7.1. Antibacterial Activity

In the dairy industry, the main bacterial pathogens that need to be controlled are those that can survive and multiply in products produced from raw milk, or that arise from contamination after pasteurization, such as *Listeria monocytogenes*, *Staphylococcus aureus, Escherichia coli*, and *Salmonella* spp. [128,129,130].

The use of LAB as starter cultures in food fermentation promotes food preservation through rapid acid production [131]. In addition to lowering pH, some LAB species/strains possess antibacterial properties resulting from a combination of factors, including competitive growth and the production of a variety of antibacterial compounds [132]. Antibacterial compounds produced by LAB include various organic acids, such as lactic acid, acetic acid, formic acid, and propionic acid, as well as such other compounds as diacetyl, acetoin, hydrogen peroxide, reuterin, and bacteriocins [133,134,135,136,137,138,139].

The efficacy of LAB to inhibit various bacterial pathogens has been demonstrated in several food matrices, including cheese, meat, and vegetables [140]. In fermented milk, the application of a bacteriocin-producing strain of *Lc. lactis* ssp. *lactis* reduced *L. monocytogenes* contamination to undetectable levels [141]. Several other studies have also shown a reduction in *L. monocytogenes* in various cheeses by using *Lc. lactis* strains that produce bacteriocins [17,142]. Such bacteriocin-producing LAB species as *L. plantarum*, *Streptococcus* spp. and *Enterococcus* spp. have been shown to reduce *L. monocytogenes* and *S. aureus* contamination in various dairy products [17,128,143,144,145,146].

### 7.2. Antifungal Activity

Molds and yeasts are ubiquitous contaminants of dairy products, especially under conditions that favor their growth [130,136]. In the case of cheese, fungal contamination occurs in all types of cheese, although more readily in soft and unripened cheeses [147,148].

Fungal spoilage causes visible or invisible sensory defects in cheese, such as the visible growth of the fungus on the surface, and the production of metabolites that lead to noticeable and unpleasant changes in aroma, flavor, and texture, thus resulting in a loss of product quality [130,135,148,149,150].

In addition to the major economic losses associated with spoilage, some fungi pose a threat to food safety due to their ability to produce mycotoxins [139,148,151,152,153,154,155]. Therefore, the risk of mycotoxins in cheese increases when toxigenic fungi are allowed to grow during production and storage [148]. Filamentous fungi belonging to the genera *Aspergillus*, *Fusarium*, and *Penicillium* can grow on the cheese surface and produce mycotoxins that are highly toxic [133,138,150,156,157,158]. Some mycotoxins are present only in the fungus, while most are excreted in food [159]. Aflatoxins are considered one of the most important and well-known classes of mycotoxins in food [159,160]. These compounds have numerous and diverse toxic properties, including carcinogenic, teratogenic, mutagenic, nephrotoxic, hepatotoxic, neurotoxic, immunosuppressive, and estrogenic effects, even when ingested at low concentrations [133,135,148,160].

Some LAB species/strains have shown activity against common cheese spoilage molds [134]. The antifungal activity of LAB is attributed to multiple compounds acting individually or in synergy to provide multiple barriers against spoilage molds [134,152]. Some LAB species are also able to reduce mycotoxins produced by molds [158].

The LAB best known for their ability to prevent or retard the growth of toxinogenic fungi belong to the genera *Lactococcus* and *Lactobacillus* and, to a lesser extent, *Pediococcus* and *Leuconostoc* [133]. The antifungal activity of the genus *Lactobacillus* has been extensively studied, with particular emphasis on the species *L. plantarum* [133,150]. Different strains of *L. plantarum* and its metabolites have been tested in a variety of foods, where they were able to inhibit various fungal species belonging to the genera *Aspergillus*, *Penicillium*, *Rhizopus*, and *Rhodotorula* [150,153]. In addition to *L. plantarum*, other species such as *L. casei, L. paracasei*, and *L. brevis* have also shown antifungal activity against a broad spectrum of spoilage molds [149].

### 7.3. Antimicrobial Metabolites Produced by LAB

#### 7.3.1. Organic Acids

The antimicrobial activity of LAB is associated with the production of organic acids, mainly lactic and acetic acids, but also formic, propionic, butyric, phenyllactic, hydroxy-phenyllactic, and indole-3-lactic acids, among others [160]. The most extensively studied acids are lactic, acetic, propionic, and phenyllactic acids [152].

Organic acids lower pH and create unfavorable conditions for the growth of many potentially pathogenic microorganisms [160]. In addition to their effects on pH, the undissociated form can diffuse across the cell membrane of the target organism, dissociate within the cell, and lower the cytoplasmic pH. Therefore, the most important parameter that determines the antimicrobial activity of an organic acid is pKa, because when pH < pKa, the undissociated form enters the cell and consequently neutralizes the electrochemical proton gradient, leading to the death of the susceptible organisms [133,135,161].

Similar to lactic acid, acetic and propionic acids interact with cell membranes to neutralize the electrochemical proton gradient; however, the effect of these acids is often dependent on the pH reduction achieved [161].

Phenyllactic acid has been described as an antimicrobial compound that exhibits a broad spectrum of antibacterial and antifungal activities [162]. This acid contributes to microbial inhibition in synergy with other compounds produced by LAB [161,162]. Phenyllactic acid can retard the growth of many fungi, including species belonging to the genera *Aspergillus, Fusarium*, and *Penicillium*. However, many studies have reported that very high concentrations of this acid are required to inhibit fungal growth, thus making it less suitable as antifungal agent in foods [163].

#### 7.3.2. Hydrogen Peroxide

Hydrogen peroxide (H_2_O_2_) is produced by most LAB in the presence of oxygen [133,135]. Since LAB are unable to produce catalase, they cannot degrade hydrogen peroxide, so it accumulates in the medium, where it exerts a strong oxidizing effect on the lipid membrane, while destroying the basic molecular structures of the cell proteins of the target organisms [133,135,161].

The bactericidal action of hydrogen peroxide has been shown to be effective in reducing spoilage bacteria and pathogens such as *E. coli, L. ivanovii*, and *S. aureus* [164].

#### 7.3.3. Diacetyl

Diacetyl (also known as 2,3-butanedione) is an aromatic compound, characterized by its buttery taste when associated with dairy products [165]. Diacetyl is produced by some LAB strains during citrate fermentation and is present in many dairy products such as cheese [122,166]. Diacetyl has been shown to exert antifungal and antibacterial effects at low pH [167,168]. However, the amounts of diacetyl required to exert antimicrobial activity significantly alter the taste and flavor of the final product [161].

#### 7.3.4. Reuterin

Reuterin was first described as produced by *L. reuteri*, and is an antimicrobial compound with a broad spectrum of activity [152,161]. It consists of acrolein and 3-hydroxypropionaldehyde (3-HPA), which can be further metabolized to 1,3-propanediol and 3-hydroxypropionic acid (3-HP) [169]. This low molecular weight compound is capable of inhibiting the growth of a wide range of microorganisms, and is one of the most intensively studied antifungal compounds [161,162].

Reuterin is produced by several LAB under anaerobic conditions via the fermentation of glycerol [138,152,161]. The main LAB producers of reuterin are lactobacilli, including the species *L. reuteri, L. brevis, L. buchneri, L. collinoids,* and *L. coryniformis* [133].

Gram-positive bacteria are generally more resistant to reuterin than Gram-negative strains, including common food pathogens such as *E. coli, Salmonella*, and *L. monocytogenes* [169]. In target organisms, reuterin can suppress ribonuclease activity [133,138] or induce oxidative stress by modifying thiol groups in proteins and glutathione [170]. In fungi, reuterin inhibits the growth of species belonging to the genera *Fusarium, Aspergillus*, and *Penicillium* [133,138,171].

#### 7.3.5. Fatty Acids

Fatty acids may also possess antibacterial and antifungal activity. The length of the fatty acid chain appears to play an important role in antimicrobial activity, with lauric (C12) and capric (C10) acids showing the best antimicrobial results [172].

LAB can produce several types of fatty acids that improve the sensory quality of fermented products. Caproic acid is one of these fatty acids and it has strong antifungal activity. It can act synergistically with propionic, butyric, or valeric acid [138].

According to Crowley et al. [162], antifungal fatty acids cleave the lipid bilayers of fungal membranes, thus causing a loss of membrane integrity. The increase in fluidity increases membrane permeability, leading to the uncontrolled release of electrolytes and intracellular proteins, as well as the cytoplasmic disintegration of fungal cells.

Some strains of lactobacilli can produce hydroxylated fatty acids from linoleic acid [173]. Sjogren et al. [174] found that hydroxylated fatty acids possess strong antifungal activity against a broad spectrum of yeasts and molds.

#### 7.3.6. Cyclic Dipeptides

Cyclic dipeptides include several types of diketopiperazines such as the 2,5-diketopiperazines, which are among the most abundant peptide derivatives in nature [162]. They can be formed in foods by chemical reactions during thermal processing, or by yeast and LAB during fermentation [175].

Several bioactive properties are attributed to these dipeptides, including antimicrobial and antitumor activities [162]. The broad spectrum of antimicrobial effects of cyclic dipeptides produced by LAB has been demonstrated in several studies [134,176,177].

#### 7.3.7. Bacteriocins

In recent years, bacteriocins have attracted considerable interest as a safe alternative to chemical preservatives for being rapidly hydrolyzed in the human gastrointestinal tract [178,179,180,181,182].

Bacteriocins are peptides with antimicrobial activity, synthesized by bacteria in ribosomes. These peptides often exhibit a narrow inhibitory spectrum and inhibit taxonomically-close bacteria [143,183,184,185]. The most common mechanisms used by bacteriocins to kill other microorganisms include the formation of pores in the cell membrane or the inhibition of cell wall synthesis [186]. Most bacteriocins produced by LAB, especially those that inhibit Gram-positive bacteria, exert their antimicrobial effects by forming pores in the membrane of target cells, thereby depleting the transmembrane potential and/or pH gradient, which eventually leads to loss of cell contents [187,188].

Bacteriocins are produced by only a few strains of different bacterial species, including LAB [189]. Some of these bacteriocins are effective against important foodborne pathogens, such as *L. monocytogenes, S. aureus, Pseudomonas aeruginosa*, and *Salmonella enterica,* as well as other spoilage microorganisms [178,189,190,191,192]. Some studies have shown that LAB can also produce bacteriocins with antifungal activity. Although *Lactococcus, Streptococcus,* and *Pediococcus* have been reported to produce bacteriocin-like peptides against a variety of fungi, *Lactobacillus* strains have been most commonly associated with the production of antifungal peptides/proteins [162]. However, the mode of action of protein compounds in inhibiting fungal growth by LAB is not completely clear [133,152,162].

The only bacteriocins commercially available at present are nisin A, produced by *Lc. lactis*, and pediocin, produced by *P. acidilactici* [51,185]. Nisin has a broad spectrum of antimicrobial inhibition, and inhibits the growth of most Gram-positive bacteria that contaminate food, such as *L. monocytogenes, S. aureus*, and *Clostridium perfringens* [193]. However, the efficacy of nisin has some limitations, since it cannot be used in foods with neutral or alkaline pH, or in foods that require LAB for fermentation [194]. Other bacteriocins, such as enterocins, have been shown to be more effective than nisin in inhibiting *L. monocytogenes* [194]. Bacteriocins that are effective against this bacterium are important for use in foods, especially cheeses produced from raw milk, as they may be contaminated with this pathogen [195].

Bacteriocins produced by LAB are often active over a wide pH range, resist high temperatures, and inhibit the growth of a variety of food spoilage and pathogenic bacteria. In addition, bacteriocins are sensitive to digestive proteases such as pancreatin, trypsin, and chymotrypsin, and therefore have no negative effects on the gut microbiota [194]. Since they are not toxic to eukaryotic cells and become inactive toward proteolytic enzymes (e.g., digestive proteases), bacteriocins are generally considered safe substances [196,197].

## 8. Probiotic Potential of LAB

Consumers are becoming increasingly aware of the beneficial effects of probiotics, and this has led to greater demand for probiotic products worldwide [198,199]. Most microorganisms residing in the gastrointestinal tract are harmless or otherwise beneficial to the host, thus resulting in a generally harmonious and symbiotic relationship [200]. The potential benefits of consuming probiotics are primarily due to positive changes in the gut microbiota, known to play a key role upon the immune system [201].

In 2002, the Food and Agriculture Organization of the United Nations (OAA) and the World Health Organization (WHO) defined probiotics as “live microorganisms that, when ingested and administered in sufficient quantities, have health benefits for the host” [202]. Therefore, probiotics are preparations of viable and non-pathogenic microorganisms included in foods or dietary supplements that interact directly with the gastrointestinal microbiota and immune system, so as to produce health-promoting effects [203,204]. According to these definitions, a large number of LAB strains have been proposed as probiotics [202,204].

In addition to modulating the immune system, the positive health effects of taking probiotics include: the improvement of lactose tolerance and digestion [199,205], the prevention and treatment of gastrointestinal infections [206], the prevention of colorectal cancer [203,207], reduction in blood cholesterol levels [208,209,210], and the improvement of mental health via the gut-brain axis [211].

Probiotic LAB strains used in the production of fermented foods or pharmaceuticals must be recognized as safe for human use and possess GRAS or QPS status [201,212]. Probiotic microorganisms must not only fulfill safety aspects, but also have functional and technological properties that are of interest. These include ease of propagation and incorporation in food, long-term survival, and clinically valid and documented beneficial health effects [201]. The safety and efficacy of probiotics must be scientifically proven in advance for each strain and product [213].

### 8.1. LAB Used as Probiotics

Most microorganisms currently recognized as probiotics belong to the LAB group [201,202,214]. This is not at all surprising, because LAB are part of the natural microbiota of the healthy gastrointestinal tract of humans and animals [215,216].

A large amount of LAB, which can be classified as probiotics, are also present in milk and fermented dairy products, such as cheese, yogurt, and fermented milk [217,218,219]. As mentioned earlier, LAB can ferment various sugars and produce organic acids such as lactate and acetate, as well as other antimicrobial metabolites such as hydrogen peroxide and bacteriocins, all of which can effectively inhibit the growth of pathogenic organisms in the gut [205,220].

Species belonging to the genera *Lactobacillus* and *Bifidobacterium* are most commonly used as probiotics because they play a very important role in maintaining proper intestinal function and stimulating the host immune system [212,221,222]. Other genera with species that exhibit probiotic properties include *Pediococcus*, *Lactococcus*, and *Enterococcus* [48,79,223].

The most commonly used probiotic lactobacilli species in the food industry are *L. acidophilus*, *L. plantarum, L. rhamnosus, L. paracasei, L. casei, L. gasseri, L. johnsonii, L. reuteri* [216,221], *L. fermentum, L. salivarius* [212], and *L. delbrueckii* subsp. *bulgaricus* [201]. As for the genus *Bifidobacteria*, the most common species in food applications are: *B. adolescentis, B. animalis* subsp. *lactis, B. bifidum, B. breve, B. longum* subsp. *longum*, and *B. longum* subsp. *children* [212].

### 8.2. Mechanisms of Action of Probiotics

The mechanisms of action may vary from one probiotic strain to another, but in most cases a combination of activities is likely, making the study of the responsible mechanisms a difficult and complex task [1]. Furthermore, the response to probiotic treatment may be specific to each individual. Several studies have shown that the gut microbiota can influence the expected effect of treatments, as it may vary greatly from person to person [224].

**Figure 2:**
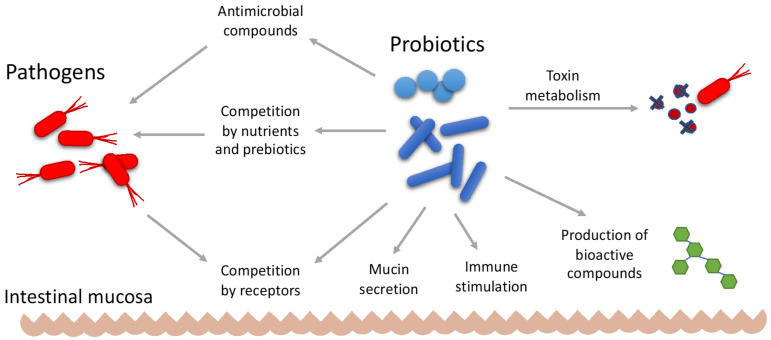
Mechanisms of action of probiotics.

Several mechanisms of action have been proposed for the therapeutic effect of probiotics, as shown in Figure 2. Probiotics may be active in preventing gastrointestinal infections by making it more difficult for pathogens to colonize the gastrointestinal tract, either by competing for nutrients or by competing for receptors. In this case, probiotics compete for a limited number of receptors on the surface of the intestinal epithelium [224,225]. The release of antimicrobial compounds such as organic acids, hydrogen peroxide, and bacteriocins may also exert antagonistic effects against pathogenic organisms [226,227].

Probiotics may also act by strengthening and increasing the intestinal mucosal barrier. Increased mucin secretion enhances the binding of probiotics to the intestinal mucosa. This effect competitively prevents the binding of enteropathogens to the receptors of the epithelium. The stabilization of the intestinal barrier permeability limits pathogen colonization, eliminates foreign antigens that have invaded the mucosa, and regulates antigen-specific immune responses [224,225]. The use of appropriate strains of probiotics may be helpful in eliminating bacteria associated with colorectal cancer, thereby reducing the risk of developing this disease. Some studies have shown promising results regarding the use of probiotics as a prevention strategy for colorectal cancer; however, clinical trials are still needed to demonstrate this therapeutic effect [228].

The modulation of the host immune system represents another form of probiotic effect. Some LAB strains can modulate innate and acquired immune responses by binding to specific receptors on immune cells and other tissues such as intestinal epithelial tissue, and by stimulating the production of cytokines, T cells, the activation of dendritic cells and macrophages, and the production of specific antibodies [229,230,231].

With the increasing recognition of the importance of healthy gut microbiota in the development of autoimmune diseases, many studies have focused on the immunomodulatory effects of some probiotic strains [230,232,233]. In one of these studies, a *Ln. citreum* strain isolated from an artisanal cheese was shown to have an immunomodulatory effect due to its ability to decrease the production of proinflammatory cytokines (IL-8) by intestinal cells [231]. In animal studies, oral ingestion of this bacterium in an asthma model (nasal administration of an allergen) resulted in immune tolerance to the allergen [231].

Probiotic LAB may also be involved in the synthesis of neurotransmitters and neuromodulators. For example, certain species of *Lactobacillus* and *Bifidobacterium* produce γ-aminobutyric acid (GABA), *Streptococcus* spp. and *Enterococcus* spp. produce serotonin, and *Lactobacillus* spp. produce acetylcholine [234,235]. The gut microbiota is also involved in modulating the expression of neurochemical receptors and modulating the brain-gut axis, leading to psychotropic, antidepressant, and anxiolytic effects [236]. Several studies in animal models have unfolded the therapeutic effect associated to the administration of probiotic LAB strains upon cognitive processes and a reduction in psychophysiological markers of anxiety and depression [237]. As a result of the potential effect of probiotics on improving mental health, the term “psychobiotics” has been proposed [238]. Psychobiotics refer to a group of probiotics that are able to produce and release neuroactive substances such as GABA and serotonin. These act through the brain-gut axis, and exert antidepressant effects by altering emotional, cognitive, and neuronal indices [236,239].

### 8.3. Bioactive Compounds Produced by Probiotic LAB

Probiotics can increase the availability of nutrients and produce bioactive soluble factors (byproducts of metabolism) that are beneficial to the host and are referred to as postbiotics [240].

Fermented dairy products, especially cheese, may contain substances that have beneficial effects on human health [29,32]. In the last decade, fundamental studies have opened a new field of research dealing with bioactive compounds from food. Bioactive compounds are components of ready-to-eat foods that can exert a regulatory effect in the human body, regardless of their nutritional function [241].

The proteolysis of milk proteins by LAB during milk fermentation and cheese ripening can result in peptides with bioactive properties that confer immunostimulatory, opioid, or angiotensin I-converting enzyme (ACE) inhibitory activity [242]. Numerous studies have shown that milk fermented by *Lactobacillus* spp. can exert beneficial effects in controlling cardiovascular disease caused by hypertension via the production of ACE-inhibitory peptides [243].

**Figure 3:**
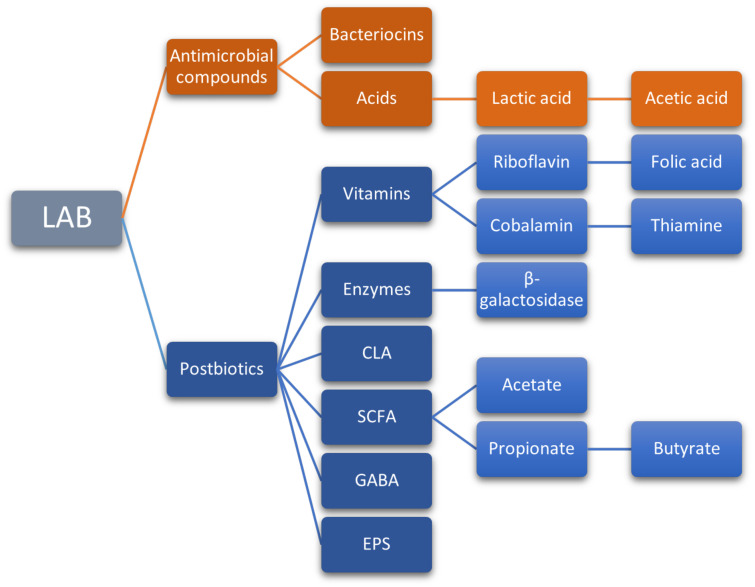
Main bioactive compounds produced by probiotic LAB.

Bioactive compounds produced by probiotic microorganisms also include vitamins (thiamine, riboflavin, cobalamin, folic acid, and vitamin K), enzymes (lactase or β-galactosidase), bioactive peptides (from the hydrolysis of proteins), conjugated linoleic acid (CLA), short-chain fatty acids (SCFA), gamma-aminobutyric acid (GABA), exopolysaccharides (EPS), and antimicrobial compounds such as bacteriocins (Figure 3) [32,244]. Some of these compounds stand out for their potential, yet poorly studied effects on human health.

#### 8.3.1. Bacteriocins

In addition to the use of bacteriocins as antimicrobial agents in foods (see Section 7.3.7), recent studies have investigated the effect of bacteriocin production by bacteria in the gastrointestinal tract. In this context, several studies on bacteriocins have focused on the treatment of infections caused by antibiotic-resistant bacteria [244,245]. The widespread and excessive use of antibiotics has deleterious effects on the gut microbiota and leads to an increase in gastrointestinal infections. Several in vitro and in vivo studies have shown that bacteriocins produced by LAB can exert a protective effect on the gastrointestinal tract by excluding pathogens and promoting the colonization of the gut [245]. Therefore, the anti-infective effect of bacteriocins produced by LAB represents a promising alternative to the use of antibiotics, especially in special cases where other methods are not allowed (e.g., pregnancy) [246]. In addition, some bacteriocins that are toxic to foodborne pathogens are often inactive to microorganisms that are beneficial to humans and do not disrupt the natural balance in the gut ecosystem [140]. Therefore, the use of bacteriocin-producing probiotic bacteria may prove an effective therapeutical approach to fight colonization of the gut by multidrug-resistant bacteria without disrupting the native microbiota [247].

Several studies have shown that the use of bacteriocin-producing bacteria is more effective than purified bacteriocins in improving gut health [186]. This is likely due to the fact that purified bacteriocins are degraded by various proteolytic enzymes during passage through the gastrointestinal tract. However, bacteriocins produced by probiotic bacteria in the gut can interact directly with pathogens. In this way, bacteriocins can be produced by probiotic bacteria in situ in the gut to combat intestinal infections [187].

Other studies have also suggested the use of bacteriocins as potential anticancer agents [244,248]. Some bacteriocins have been shown to exert selective effects on cancer cells, probably due to their unique membrane composition [249].

#### 8.3.2. Short Chain Fatty Acids (SCFA)

Presently, there is a wealth of evidence that short-chain fatty acids (SCFA) produced by gut microorganisms during the fermentation of partially digestible and indigestible foods are involved in the prevention of some chronic diseases [250]. Short-chain fatty acids include mainly acetate (ethanoic acid, C2:0), propionate (propanoic acid, C3:0), and butyrate (butanoic acid, C4:0). These SCFAs are produced by some intestinal microorganisms, such as *Clostridium*, *Bacteroides*, *Lactobacillus*, and *Bifidobacterium* from the fermentation of dietary fiber, resistant starch, oligosaccharides, and other compounds that are not directly digested by intestinal digestive enzymes [250].

SCFAs are estimated to account for ~60–70% of the energy requirements of colon epithelial cells and 5–15% of the total caloric requirements of humans [251]. These fatty acids, especially butyrate, have shown therapeutical potential in various diseases such as inflammatory bowel disease, antibiotic-associated diarrhea, and colon cancer [252].

Studies on the human gut microbiota have shown that fewer butyrate-producing bacteria are found in stool samples from patients with type 2 diabetes than in healthy controls, thus suggesting a possible protective role of butyrate in obesity-related metabolic diseases [251]. There is a growing body of evidence that butyrate also has effects on the brain via the gut-brain axis [253]. For example, butyrate may increase the proportion of cholinergic enteric neurons through epigenetic mechanisms. Through its ability to cross the blood-brain barrier, butyrate activates the *vagus* nerve and hypothalamus, which may influence appetite and eating behavior [254].

Other studies have shown that SCFA, particularly acetate and butyrate, can act as anti-inflammatory agents and have been shown to be effective in inhibiting the production of pro-inflammatory cytokines and maintaining intestinal barrier immune function [255]. However, one of the best-studied effects of SCFA relates to the incidence and development of colorectal cancer. A high-fiber, low-fat, low-protein diet can effectively increase the concentration of SCFAs in the intestinal tract. SCFAs promote apoptosis and inhibit cancer cell proliferation by inducing epigenetic changes such as methylation and deacetylation, thus triggering T cell-mediated immune responses, and activating intracellular signaling pathways [250].

#### 8.3.3. Conjugated Linoleic Acid (CLA)

Conjugated linoleic acid (CLA) is a mixture of positional and geometric isomers of linoleic acid (octadecadienoic acid, C18:2, cis-9, cis-12) with a system of conjugated double bonds [256]. The isomers cis-9, trans-11 (c9, t11), trans-10, cis-12 (t10, c12), and trans-9, trans-11 (t9, t11) have received particular attention due to their remarkable biological activities [18].

Several health benefits have been attributed to CLA, and there is increasing evidence that these effects are isomer-specific [257]. The cis-9, trans-11 (c9, t11) isomer is the most abundant and the most frequently associated to beneficial health effects [258]. It is incorporated into the phospholipids of cell membranes and exerts its effects particularly on arachidonic acid metabolism [256]. The trans-10, cis-12 (t10, c12) isomer has been associated with changes in body composition and sometimes linked to inflammatory responses in adipose tissue (Gong et al., 2019). This isomer is known to have the most potent effect of CLA in preventing cell proliferation and inducing apoptosis in cancer cells [256]. The trans-9, trans-11 C18:2 isomer also has potent growth inhibitory and antiproliferative effects on the growth of human colon and breast cancer cells [259,260].

CLA has several functional properties and potential health-promoting effects, such as anticarcinogenic, anti-inflammatory, reducing and preventing body fat deposition, reducing atherosclerosis [261,262], modulating the immune system [263], reducing blood glucose levels [264], reducing osteoporosis [265], preventing and treating cardiovascular disease, controlling serum levels of cholesterol and triglycerides, and improving insulin resistance [256].

CLA is found in many foods, in greater amounts in milk, dairy products, and beef, and in lesser amounts in pork, poultry, and vegetable oils [264]. The amount ingested through the diet is far from sufficient to achieve the desired effect. One of the most effective methods to increase CLA intake in humans is to produce foods containing strains with a high potential for CLA production [266]. Alternatively, CLA can be produced by the gut microbiota, or by probiotic bacteria in the diet that can utilize dietary linoleic acid toward CLA [259,266].

In recent years, several studies have shown that some strains of LAB and bifidobacteria can efficiently convert linoleic acid to CLA, due to the activity of the enzyme linoleic acid isomerase; however, this is a strain-dependent process [263]. Some genera such as *Lactobacillus*, *Propionibacterium*, *Bifidobacterium*, *Enterococcus* [256,262], *Leuconostoc*, and *Lactococcus* [258,267] produce CLA in synthetic media or in milk. However, the ability to produce CLA is again strain-dependent [256,262].

#### 8.3.4. Gamma-Aminobutyric Acid (GABA)

γ-Aminobutyric acid (GABA) is a non-protein amino acid that is widely distributed in microorganisms, plants, and animals [268,269,270]. GABA is synthesized by glutamate decarboxylase (GAD), which catalyzes the irreversible α-decarboxylation of L-glutamate or its salts to GABA [271]. GAD is the key enzyme for the bioconversion of GABA and uses pyridoxal-5′-phosphate for cofactor [272].

GABA is the major inhibitory neurotransmitter of the central nervous system [269,272,273,274]. GABA has several important physiological functions, such as blood pressure regulation, diuretic effects, and insulin secretion; hence, it may contribute to the prevention of diabetes [269,275].

Some studies demonstrate a positive effect of GABA in the treatment of insomnia [276], depression [277], and chronic symptoms associated with alcohol problems [269]. Other functions attributed to GABA include relaxation, relief of anxiety, anti-inflammatory effects, control of asthma, and improvement of oxidative stress [269,272,278,279,280]. GABA intake may also help in the treatment of various neurological disorders, such as Parkinson’s disease, dementia, seizures, and schizophrenia [281]. GABA also regulates growth hormone secretion and shows antiproliferative activity in colon cancer cells [274]. This bioactive compound is further involved in the regulation of heart rate and hormone secretion and has even been used to improve memory [282,283,284,285]. There is scientific evidence of GABA’s effect on regulating thyroid hormones and improving thyroid function, with implications for regulating obesity [286,287,288,289,290]. However, the best documented effect of GABA is the regulation of blood pressure by acting as an antihypertensive agent [291].

Numerous studies have demonstrated the presence of GAD in some strains of LAB [270,292]. Although GAD is widely distributed in LAB, the ability to produce GABA varies greatly among species and strains [278]. LAB from food sources has been shown to hold a greater ability to produce GABA [274,293]. Fermented foods rich in L-glutamate are important sources for the isolation of GABA-producing LAB [272]. Since caseins are rich in L-glutamate and are released by proteolytic enzymes during fermentation, decarboxylation of this amino acid to GABA may have an important effect on its concentration in cheese [241,275]. Indeed, strains of *L. buchneri, L. brevis, L*. *paracasei, L. plantarum*, and *Lc. lactis* isolated from traditional cheeses have shown a high capacity for GABA production [269,275].

Many LAB species/subspecies have shown the capacity to produce GABA, although the amount produced varies greatly. Among lactobacilli, there are numerous GABA-producing species, such as *L. brevis, L. buchneri, L. delbrueckii ssp. bulgaricus, L. fermentum, L. helveticus, L. paracasei,* and *L. plantarum* [272,276,294,295]. In addition, some strains of *S. salivarius* subsp. *thermophilus* and *Lc. lactis* can produce GABA [272]. In recent years, some species of the genera *Enterococcus*, *Leuconostoc*, *Pediococcus*, *Propionibacterium*, and *Weissella* have been found to be capable of producing GABA [272].

The production of GABA by microorganisms is influenced by several factors, namely initial pH, L-glutamic acid concentration, temperature, fermentation time, and culture medium additives such as carbon and nitrogen sources [272,292]. The pH is a key factor for GABA biosynthesis by LAB and affects not only bacterial growth but also the activity of GAD [272]. Although the GAD properties of different species and strains vary widely, most GADs show optimal activity at pH 4.0–5.0.

Dietary intake of GABA is relatively low and requires the consumption of products fortified with this compound [269]. Strategies to increase GABA levels in humans include the consumption of GABA-enriched foods. Alternatively, GABA can be synthesized by the gut microbiota via ingestion of probiotic bacteria, which have a high capacity to colonize the gastrointestinal tract and produce GABA in situ [274]. There are several reports on the production of LAB-fermented products that can accumulate high amounts of GABA [296,297]. Consequently, GABA-producing LAB can be used for the development of health-oriented fermented foods.

#### 8.3.5. Exopolysaccharides (EPS)

Microbial exopolysaccharides are extracellular polymers composed of linear or branched chains that may differ in monosaccharide composition and degree of branching [298].

Some LAB species are able to synthesize and excrete extracellular polysaccharides [14,299]. Depending on their chemical composition, EPS are classified as homopolysaccharides (HoPS) when they contain a single type of monosaccharide, and heteropolysaccharides (HePS), which comprise repeating units of different monosaccharides [300,301]. HoPs can consist of linear or branched chains of glucose or fructose and are classified as α-D-glucans (e.g., dextran, mutan, alternan, and reuteran), β-D-glucans, fructans (e.g., levan and inulin), and polygalactans (Torino et al., 2015). HePs may consist of D-glucose, D-galactose, L-rhamnose, mannose, arabinose, or fucose. In some cases, N-acetylglucosamine, N-acetylgalactosamine, and other monosaccharides such as fucose and ribose are also present [302]. Gellan, xanthan, and kefiran are examples of HePs. Unlike HoPS, the constituent monomers of HePS are synthesized intracellularly but polymerized outside the cell [302].

EPS production by LAB occurs in the presence of excess substrate (available sugar such as sucrose) or under growth-limiting conditions due to the lack of essential elements such as nitrogen, phosphorus, sulfur, or magnesium [303]. However, the EPS produced by LAB depend not only on the culture conditions and medium composition, but also on the particular strain [304]. The presence of EPS associated with bacterial cells is detected in solid media by the formation of mucoid colonies, and in liquid media by the increase in viscosity [303].

In the food industry, EPS produced by LAB are used as stabilizers, emulsifiers, and gelling agents to modify the rheological properties and texture of products [300,304]. These bacterial EPS are normally used as food additives, but LAB cultures can also produce these EPS in situ during lactic acid fermentation. For this reason, the use of EPS as bio-thickeners for food has received considerable attention [300]. Moreover, EPS produced in situ by LAB in the intestine may exert a prebiotic effect to promote colonization by probiotic bacteria, such as lactobacilli and bifidobacteria [14].

Most EPS-producing LAB belong to the genera *Streptococcus*, *Lactobacillus*, *Lactococcus*, *Leuconostoc*, *Pediococcus, Enterococcus*, and *Weissella* [298,300,301]. Some strains of the genus *Bifidobacterium* are also capable of producing these biopolymers [298,300].

EPS exhibit various health-promoting effects such as antitumor, antioxidant, immunomodulatory, and prebiotic effects [231,299]. EPS produced by probiotic LAB may also affect the gastrointestinal tract by protecting intestinal cells from toxins and lowering cholesterol levels by increasing bile acid excretion [299,305,306]. Several studies have reported other beneficial effects of EPS produced by LAB, including the control of blood glucose levels, the absorption of calcium and magnesium, and antioxidant effects [299,307].

## 9. Conclusions

Lactic acid bacteria (LAB) added initially or as part of the natural microbiota of milk play an important role as starter cultures for the manufacture of traditional raw milk cheeses. In the secondary cheese microbiota, LAB contribute to the maturation of cheese and influence the texture, flavor, and aroma of the final product. The preservative effect of LAB results chiefly from the formation of primary metabolites such as lactic and acetic acids, hydrogen peroxide, and bacteriocins. Moreover, indigenous bacteriocin-producing LAB can be explored as efficient alternatives for food preservation. Recent studies using the HTS methodologies have contributed to a better knowledge of the traditional cheese microbiota, which might result in the application of improved LAB starter cultures and cheesemaking practices to produce more consistent and higher quality cheeses.

On the other hand, knowledge of the benefits of the intestinal microbiota for many physiological processes of the host has opened new possibilities for the application of certain LAB strains as probiotics. Current scientific evidence suggests that LAB, mainly *Lactobacillus* and *Bifidobacterium*, are beneficial to the host in correcting imbalances in the intestinal microbiota, and consequently in maintaining and regulating health. These bacteria are traditionally associated with fermented foods and are the most studied probiotic organisms. Probiotic organisms can protect the host from intestinal disease by inhibiting toxin production, producing antibacterial compounds, blocking pathogen adhesion sites, competing for nutrients, and stimulating immunity. In addition to pathogen exclusion, probiotics may offer other beneficial properties to the host’s health, including nutrient synthesis (certain vitamins), reduction in lactose intolerance, and production of bioactive compounds such as CLA, SCFA, and EPS. More recently, evidence has emerged that probiotics, referred to as psychobiotics, can influence the gut-brain axis and thus promote mental health. Further in vitro and in vivo studies are still necessary to demonstrate the human health benefits derived from consumption of traditional cheeses, though.

## Data Availability

Not applicable.
